# A Deep Learning Model to Automate Skeletal Muscle Area Measurement on Computed Tomography Images

**DOI:** 10.3389/fonc.2021.580806

**Published:** 2021-05-07

**Authors:** Kaushalya C. Amarasinghe, Jamie Lopes, Julian Beraldo, Nicole Kiss, Nicholas Bucknell, Sarah Everitt, Price Jackson, Cassandra Litchfield, Linda Denehy, Benjamin J. Blyth, Shankar Siva, Michael MacManus, David Ball, Jason Li, Nicholas Hardcastle

**Affiliations:** ^1^ Bioinformatics Core Facility, Cancer Research Division, Peter MacCallum Cancer Centre, Melbourne, VIC, Australia; ^2^ Sir Peter MacCallum Department of Oncology, The University of Melbourne, Parkville, VIC, Australia; ^3^ Cancer Research Division, Peter MacCallum Cancer Centre, Melbourne, VIC, Australia; ^4^ Radiation Therapy, Peter MacCallum Cancer Centre, Melbourne, VIC, Australia; ^5^ Institute for Physical Activity and Nutrition (IPAN), Deakin University, Geelong, VIC, Australia; ^6^ Allied Health Department, Peter MacCallum Cancer Centre, Melbourne, VIC, Australia; ^7^ Radiation Oncology, Peter MacCallum Cancer Centre, Melbourne, VIC, Australia; ^8^ Physical Sciences, Peter MacCallum Cancer Centre, Melbourne, VIC, Australia; ^9^ Melbourne School of Health Sciences, The University of Melbourne, Melbourne, VIC, Australia; ^10^ Centre for Medical Radiation Physics, University of Wollongong, Wollongong, NSW, Australia

**Keywords:** deep learning, convolutional neural networks, skeletal muscle, image segmentation, sarcopenia, lung cancer

## Abstract

**Background:**

Muscle wasting (Sarcopenia) is associated with poor outcomes in cancer patients. Early identification of sarcopenia can facilitate nutritional and exercise intervention. Cross-sectional skeletal muscle (SM) area at the third lumbar vertebra (L3) slice of a computed tomography (CT) image is increasingly used to assess body composition and calculate SM index (SMI), a validated surrogate marker for sarcopenia in cancer. Manual segmentation of SM requires multiple steps, which limits use in routine clinical practice. This project aims to develop an automatic method to segment L3 muscle in CT scans.

**Methods:**

Attenuation correction CTs from full body PET-CT scans from patients enrolled in two prospective trials were used. The training set consisted of 66 non-small cell lung cancer (NSCLC) patients who underwent curative intent radiotherapy. An additional 42 NSCLC patients prescribed curative intent chemo-radiotherapy from a second trial were used for testing. Each patient had multiple CT scans taken at different time points prior to and post- treatment (147 CTs in the training and validation set and 116 CTs in the independent testing set). Skeletal muscle at L3 vertebra was manually segmented by two observers, according to the Alberta protocol to serve as ground truth labels. This included 40 images segmented by both observers to measure inter-observer variation. An ensemble of 2.5D fully convolutional neural networks (U-Nets) was used to perform the segmentation. The final layer of U-Net produced the binary classification of the pixels into muscle and non-muscle area. The model performance was calculated using Dice score and absolute percentage error (APE) in skeletal muscle area between manual and automated contours.

**Results:**

We trained five 2.5D U-Nets using 5-fold cross validation and used them to predict the contours in the testing set. The model achieved a mean Dice score of 0.92 and an APE of 3.1% on the independent testing set. This was similar to inter-observer variation of 0.96 and 2.9% for mean Dice and APE respectively. We further quantified the performance of sarcopenia classification using computer generated skeletal muscle area. To meet a clinical diagnosis of sarcopenia based on Alberta protocol the model achieved a sensitivity of 84% and a specificity of 95%.

**Conclusions:**

This work demonstrates an automated method for accurate and reproducible segmentation of skeletal muscle area at L3. This is an efficient tool for large scale or routine computation of skeletal muscle area in cancer patients which may have applications on low quality CTs acquired as part of PET/CT studies for staging and surveillance of patients with cancer.

## Introduction

Loss of skeletal muscle (SM) mass is an important consideration in oncologic patients as a key component of cancer-related malnutrition, sarcopenia and cachexia (1, 2). The loss of skeletal muscle occurring in these conditions has been linked with diminishing physical function (3, 4), increased risk of chemotherapy-related toxicities (5) and unfavorable survival outcomes (6–8). Early diagnosis and intervention with nutrition and exercise however, may improve outcomes in patients with loss of skeletal muscle (4). Importantly, weight and body mass index (BMI) alone are not good predictors of sarcopenia or cancer-related malnutrition (4, 9). Therefore, specifically in the oncology setting, there is a clear need to identify the presence of low skeletal muscle mass and intervene as necessary to reduce adverse effects.

Computed Tomography (CT) is proven to be an effective method to evaluate total body SM mass. In particular, cross-sectional area of SM at the third lumbar (L3) vertebra on abdominal CTs has been found to be highly correlated with the total body SM mass (10, 11). SM area at L3 normalized by patient height is commonly used as a surrogate marker of sarcopenia in cancer (9, 12) and as a component of recent diagnostic criteria for malnutrition and sarcopenia (2, 13). This marker is known as the L3 skeletal muscle index (SMI). Accurate segmentation of SM on CT is a time-consuming task and requires specific skill, training and experience, which limits the measurement in routine clinical practice as well as for large cohort studies. The advances in deep learning and computing resources in recent years provide novel opportunities to revisit these types of manual, time-consuming and routine tasks. In particular, deep learning has been shown to be particularly well suited to segmentation tasks (14, 15).

Previous work has demonstrated high accuracy deep learning segmentation in skeletal muscle of diagnostic quality CT scans acquired for a range of cancer and non-cancer indications (16–18). Positron Emission Tomography PET/CT studies are standard of care in staging and surveillance for a range of cancers (19–21), and are typically whole body acquisitions therefore are well suited for measurement of L3 skeletal muscle area. CT scans acquired during these studies are typically low quality, often only acquired for attenuation correction (22). These CT scans are acquired with reduced current to reduce patient dose, and no intravenous contrast, resulting in increased noise and reduced soft-tissue contrast (23). AI segmentation as trained on high quality diagnostic CT images may thus not be applicable to low quality CT scans such as those obtained in PET/CT studies. The current study aims to use a 2.5D convolutional neural network (CNN) based model to automatically segment SM area at L3 on low quality CT scans acquired as part of PET/CT studies.

## Methods

### Study Design

A CNN based deep learning model was trained to automatically segment the skeletal muscle in an axial L3 slice of a full body CT scan. Manual segmentation was performed by a single observer according to the Alberta protocol (10, 11). A data set consisting of 147 scans obtained from 66 patients were used as the training and validation set (**Table 1**). A separate data set of 116 CTs from 42 patients were used to independently test the model (**Table 1**). The accuracy of the CNN model was assessed by comparing the overlap between manual and automatic skeletal muscle contours (Dice score) and percentage error between manual and CNN based contours. Approval to conduct this retrospective study was granted by the Institutional Research and Ethics Committee.

**Table 1:** Patient and scanner information.

	Cohort 1 (Training and validation set)	Cohort 2 (Independent testing set)
No. of Patients	66	42
No. of scans	147	116
Age at first scan (mean ± SD)	66.94 ± 9.81	67.03 ± 8.72 (based on 35 patients)
Gender (female/male)	24/42	9/26 (based on 35 patients)
Slice thickness	Average 3.0 mm (range 0.6-5.0)	Average 3.3 mm (range 3.0-3.3)
PET/CT Scanner	**Nine** different scanners from **eight providers**	**Two** different scanners from **one provider**
Scanner energy	80 – 140 kV	140 kV
Manual segmentation	Observer A	Observer B

### Training and Validation Dataset

The training and validation dataset consisted of 66 patients who underwent repeat FDG PET/CT scans as part of a prospective observational trial investigating lung function PET/CT (ACTRN12613000061730). The trial protocol has been previously described (24); inclusion criteria was any patient receiving curative intent radiotherapy for NSCLC, with or without chemotherapy. Each patient had one baseline scan and up to three follow-up scans. Overall, the training dataset consists of 147 CTs (**Table 1** and **Supplementary Table 1**). Manual segmentation was performed by a single observer (observer A). This dataset will be referred to as Cohort 1.

#### Independent Testing dataset

Independent Testing Dataset The dataset was derived from a prospective observational trial of 42 patients with NSCLC, which investigated the associations between interim tumor responses on 18F-flurodeoxyglucose (18F-FDG) PET/CT and 18F-fluoro-thymidine (18F-FLT) PET/CT and patient outcomes including progression-free survival and overall survival (ACTRN 12611001283965). The methodology for the original study has previously been described (25); inclusion criteria was any patient receiving curative intent radiotherapy for NSCLC, with or without chemotherapy. The survival outcomes and the relationship between skeletal muscle loss was described in (26). There were 42 patients in the testing set with NSCLC, with a base-line scan and up to 4 follow-up scans. In total, the testing dataset consists of 116 CTs (**Table 1** and **Supplementary Table 1**). Manual segmentation was performed by a single observer (observer B). This dataset will be referred to as Cohort 2.

### Manual Segmentation

Manual segmentation of the skeletal muscle at an axial L3 slice of the full body CT scan was performed according to the Alberta protocol (10, 11). Briefly, the skeletal muscle including external and internal oblique, psoas, paraspinal, transverse abdominis and rectus abdominis was segmented using Hounsfield Unit (HU) thresholds of -29 – 150. This was manually adjusted to exclude ligaments and connective tissue around the vertebra. A single expert trained in the Alberta protocol (NK) supervised two observers to perform the segmentation according to the protocol. The training and test datasets were contoured by different observers, who performed cross comparison and review of segmentations with the expert as well as medical staff on the study (NB). Subsequent revisions of segmentations were performed if deemed necessary to adhere to the Alberta protocol. To measure inter-observer consistency and to provide inter-observer context to the automated segmentation results, the two observers each performed segmentation on 20 images of the other observer’s data, resulting in 20 images from each data set with segmentation from both observers. The inter-observer difference was computed using Dice score and absolute percentage difference.

### Neural Network Development

We experimented with several model architectures, cross-validation designs, loss functions, augmentation techniques, and optimization methods. All the models were implemented using PyTorch. The initial model consisted of a variation of 2D U-Net (27) (**Figure S1**). The model was trained by dividing the patients in Cohort 1 into two disjoint groups: training and validation. This ensures that our model is never trained and validated on scans from the same patient. The validation set had 10% of randomly selected patients. The model was trained using several loss functions including binary cross entropy loss, Dice loss (28) and focal loss (29). The trained model was evaluated using the validation set at each epoch in terms of network loss and Dice score. The model with the best average validation Dice score was retained and tested using Cohort 2. We achieved a median absolute percentage error (APE) in skeletal muscle area of 3.82% under the 2D model. The 2D model had poor performance mainly in distinguishing SM from other organs such as liver.

A 3D U-Net (30) model can improve the predictions by analyzing 3D volumes simultaneously, which mimics the manual segmentation procedure. Therefore, we tested a 2.5D U-Net architecture for SM segmentation; we have defined this as 2.5D as we have constrained the model to three axial slices, as opposed to a full 3D volume. Two setups were used to train the 2.5D model. Firstly, we used the same training and validation sets from Cohort 1 and trained one 2.5D model. Secondly, we trained an ensemble of 2.5D models. We divided the training patients (Cohort 1) into five groups, namely CV1 to CV5, to train five 2.5D models (5-fold cross validation). At each training round, we held back one group of patients as validation set and used the other four groups for the training process. This model achieved a median APE of 1.46%. Therefore, we discarded the 2D model in favor of the 2.5D model. The ensemble technique is used to tackle overfitting potential in a small sample size problem. It is expected that the five individual models may have varying performance, but can collectively give a consensus decision that outperforms traditional training.

The following sections describe in detail, the 2.5D model architecture, loss function, optimization and neural network training.

#### 2.5D U-Net Architecture

**Figure 1:**
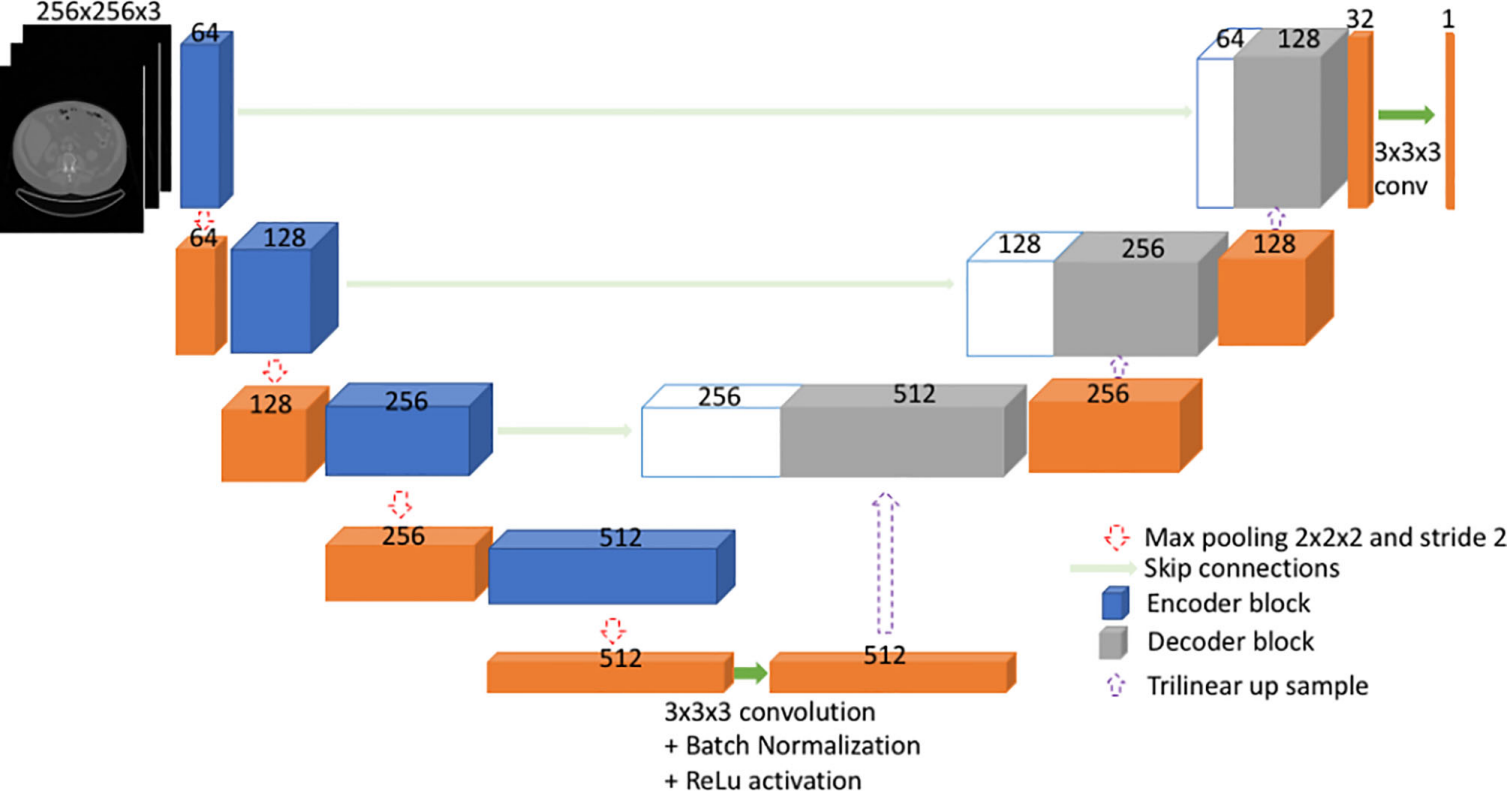
2.5D U-Net like architecture used in the current model.

U-Net (27, 30) is a type of fully convolutional neural network (FCNN). Mainly, it consists of a contracting path and an expanding path (**Figure 1**). First, the input goes through the contracting path, which consists of convolutional blocks and focuses on finer details of the image at the expense of losing spatial information. Each convolutional block in the contracting path consists of two sets of 2.5D convolutional steps, batch normalization and rectified linear unit (ReLU) activations. All convolutions are 3x3x3 with stride 1 and padding 1. Finally, a max pooling step is performed for down sampling (halving the feature set) from one encoder block to the other encoder block down the line.

Secondly, during the expanding path, the spatial information is recovered by means of skip connections (**Figure 1**). Before going through the convolutional block, the input undergoes an up-sampling step to expand dimensions. The up-sampling is done by means of trilinear interpolation. The expanding path consists of three convolutional blocks. Here, each convolution block comprises of three sets of 2.5D convolutions followed by batch normalization and ReLU. All convolutions are 3x3x3 with stride 1 and padding 1.

#### Input to the Model

The input to the model is the three axial slices from the CT scan, which consists of the L3 slice and adjacent slices on top and bottom of L3. The input is pre-processed by replacing the pixel values outside -29 to +150 HU (Hounsfield unit) range by 0. Further, input is resized to 256x256x3 from a 512x512x3 image.

#### Training the Proposed Model Architecture

Training data set consisted of CT image stacks of the three slices and manually contoured skeletal muscle at L3. To increase the number of training data, we performed data augmentation ‘on the fly’. Specifically, we performed horizontal flip, vertical flip and addition of Gaussian noise. The use of Gaussian noise is a technique to improve generalization ability of the trained model, implicitly assuming that CT images can be degraded with a Gaussian noise component. It is noteworthy that other augmentation techniques – cropping, rotation, random translation and elastic transformation – were deliberately omitted in the final model as they did not improve performance. The loss function was a combination of Dice loss (28) and focal loss (weighted cross entropy loss) (29) and model weights were optimized using “Adam” (31) optimization technique during training. The network was trained up to 300 epochs. The best model was selected based on the model’s accuracy, which was measured by Dice score, on the validation set at each epoch. We performed 5-fold cross validation (CV) and retained the best model for each fold (**Figure S2**). The entire cross-validation model training process took ~14 hours.

#### Output of the Model

The output of the model is a probability map of the image pixels. The probabilities give the model’s confidence on predicting each pixel being inside or outside of skeletal muscle area. The output, which is 256x256x3, is scaled up to match the original image dimensions of 512x512x3. Pixels with a probability above 0.3 were included in the resultant segmentation. The threshold of 0.3 was chosen empirically based on inclusiveness and absolute percentage error; 0.5 was also compared but was too stringent and led to suboptimal results. The segmentation output of the model was compared with ground truth segmentation using Dice score, absolute percentage error (APE) in skeletal muscle area and SMI. Further, the model was compared with an existing 2D deep learning segmentation model (16). The AutoMATiCA model had been trained on diagnostic quality CT scans with (mean ± SD) tube current of 338 ± 123 mA, which was substantially higher than the tube current in the validation set images (mean ± SD) 158 ± 52 mA.

## Results

### Inter-Observer Variation

Strong agreement between both observers was achieved with a mean ± SD Dice score 0.96 ± 0.02 and mean ± SD absolute percentage difference in muscle area of 2.9 ± 2.5% for the 40 images with both observers’ contours.

### Cross-Validation Performance on Cohort 1

**Figure 2:**
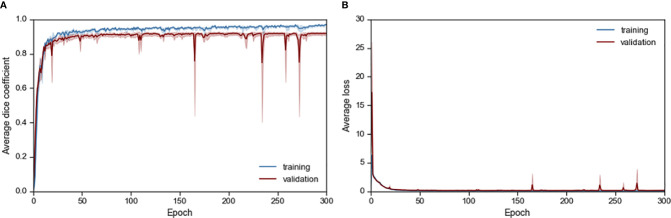
Average **(A)** Dice score and **(B)** loss performance for training and validation data during network training.

The resultant network consists of a 2.5D U-Net trained using focal loss and Dice loss for 300 epochs. The training and validation performance of Cohort 1 in terms of Dice score and network loss for the five CVs are given in **Figure S3** and **Figure S4** respectively. Average performance during CV is given in **Figure 2**. For each CV, the results from best model on validation set are given in **Table 2** and **Supplementary Table 2**. The results in terms of Dice score and percentage/absolute percentage error between manual and automatic contours are given. All CV folds show similar performance.

**Table 2:** Validation results for cross validation (CV) in terms of mean and standard deviation (SD) of performance measure.

	CV 1	CV 2	CV 3	CV 4	CV 5
**Training**					
**No. of patients**	52	53	53	53	53
**Total scans**	112	118	122	117	119
**Validation**					
**No. of patients**	14	13	13	13	13
**Total scans**	35	29	25	30	28
**Mean skeletal muscle area (cm^2^)**					
**(mean ± SD)**	122.37 ± 34.84	138.75 ± 33.33	148.54 ± 34.34	141.15 ± 35.38	130.63 ± 43.44
**[Range]**	42.05 – 178.35	83.91 – 186.35	86.55 – 213.48	80.87 – 226.47	76.81 – 205.03
**Dice score**					
**(mean ± SD)**	0.90 ± 0.07	0.91 ± 0.06	0.93 ± 0.03	0.93 ± 0.02	0.90 ± 0.04
**Percentage Error (PE) - %**					
**(mean ± SD)**	0.09 ± 9.31	-4.73 ± 4.11	-2.28 ± 3.31	-2.79 ± 4.22	-6.81 ± 5.98
**Absolute Percentage Error (APE) - %**					
**(mean ± SD)**	4.67 ± 8.02	4.78 ± 4.04	3.50 ± 1.90	3.88 ± 3.21	6.81 ± 5.98

### Ensemble Learning Outperforms Individual Learning

For each image in the test set, five different probability maps were predicted using the five models from 5-fold CV. Each image took ~0.4 seconds to go through the five models. Then, the final probability map for a test image was calculated by combining the probabilities of the five probability maps. Any pixel with probability greater or equal to 0.3 was classified as positive (i.e. belongs to skeletal muscle) and others as negative (i.e. outside of skeletal muscle).

**Figure 3:**
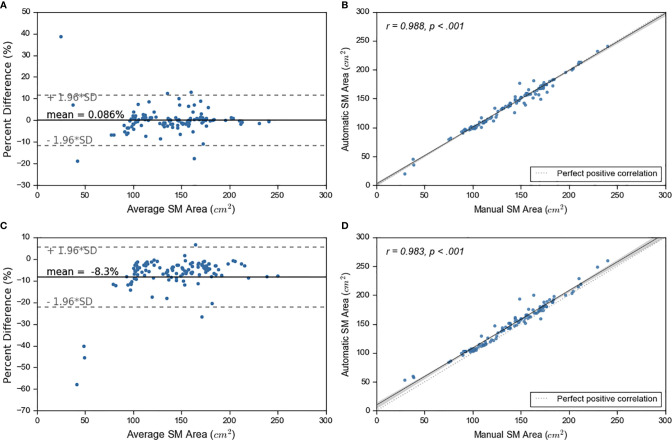
Ensemble learning results. On left, sub figures **(A, C)** show Bland-Altman plots. On right, sub figures **(B, D)** show correlation plots. The top graphs show the results for average probability and bottom graphs show results for maximum probability maps.

We experimented with two approaches on how to combine the outputs from the five models: 1) taking the average probability and 2) taking the maximum probability. We compared the SM area from both approaches to the manual SM area (**Figure 3**). Superior performance was achieved by calculating average probability maps. Based on average probability, the skeletal muscle area for the test set ranged from 19.76 cm^2^ to 241.04 cm^2^ (with mean ± standard deviation of 138.88 cm^2^ ± 38.27 cm^2^). The majority of test cases (n=92, 79%), were within ±5% error between manual and automated contours. 57 (49%) cases showed an error within ± 1% (**Figure 3**, **Figure S5**).

### Accuracy of Segmentation of Skeletal Muscle Using the Deep Learning Model

**Figure 4:**
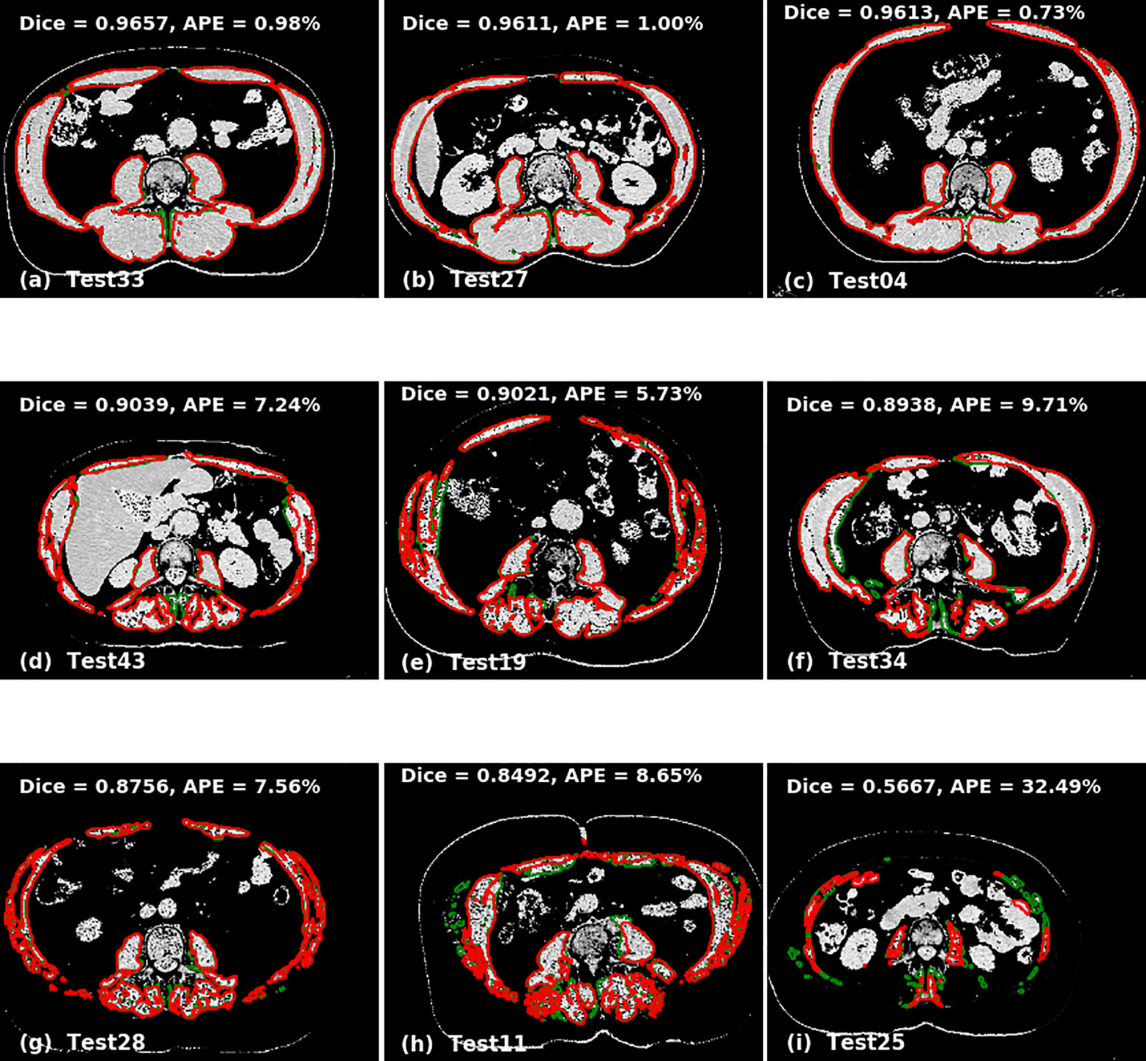
Qualitative performance of the model on Cohort 2. Red represents deep learning contours and green represents manual contours. Panels **(A–C)** in the top panel show three of the best performing cases (Dice ± 0.96). Panels **(D–F)** in the middle panel show the cases with average performance (Dice ± 0.90) and Panels **(G–I)** in the bottom panel show cases with lowest performance (Dice ± 0.88).

The accuracy of the model was calculated by Dice score and difference in area between manual and automated contours. The prediction accuracies are given in **Table 3** for 2D U-Net, single 2.5D U-Net model and the ensemble of 2.5D U-Net models (**Supplementary Table 3**). Improvements between the 2D U-Net and 2.5D U-Net was limited to classification of the liver-muscle interface; where there was no clear border between the liver and adjacent muscle on the L3 slice, the 2D U-Net was unable to accurately define the skeletal muscle. Use of the 2.5D U-Net improved this. The qualitative results are shown in **Figure 4**. Top, middle and bottom rows show representative contours from best performing, less than average performing and least performing cases respectively. **Figure 4I** shows a scan with a very low skeletal muscle area compared to all other images in training and testing sets, and this resulted in a very low Dice score. For the 20 scans in the test data set that had contours from both observers, the inter-observer mean ± SD of the dice scores was 0.96 ± 0.02 and the mean ± SD of the absolute percentage difference in muscle area was 2.7 ± 2.5%. For the same 20 scans, the mean ± SD of the dice scores was 0.93 ± 0.03 and the mean ± SD of the absolute percentage error in muscle area was 3.8 ± 2.8%, showing slightly inferior performance compared with inter-observer variation.

**Table 3:** Performance on test data set using 2.5D/2D U-Net.

Performance measure	Median	Mean ± Std. dev.	Range
**Based on 5-fold cross validation (2.5D U-Net)**			
Dice score	0.94	0.92 ± 0.06	0.57 – 0.97
APE (%)	1.46	3.09 ± 4.52	0.02 – 32.49
**Based on single model with 0.1 validation set (2.5D U-Net)**			
Dice score	0.94	0.92 ± 0.07	0.41 – 0.97
APE (%)	2.80	4.86 ± 7.62	0.04 – 54.28
**Based on single model with 0.1 validation set (2D U-Net)**			
Dice score	0.94	0.92 ± 0.05	0.57 – 0.97
APE (%)	3.82	5.16 ± 5.64	0.03 – 46.55
**AutoMATiCA results**			
APE (%)	4.0	4.98 ± 6.78	0.20 – 53.68

The AutoMATiCA model was applied to all images in the validation image set. The Dice score was not able to be computed as AutoMATiCA does not export segmentation, only the muscle area measurement and a merged image file. The mean ± SD APE for AutoMATiCA was 5.0 ± 6.8%, compared with 3.1 ± 4.5% for the current model.

### Using Model Output to Predict Sarcopenia

**Figure 5:**
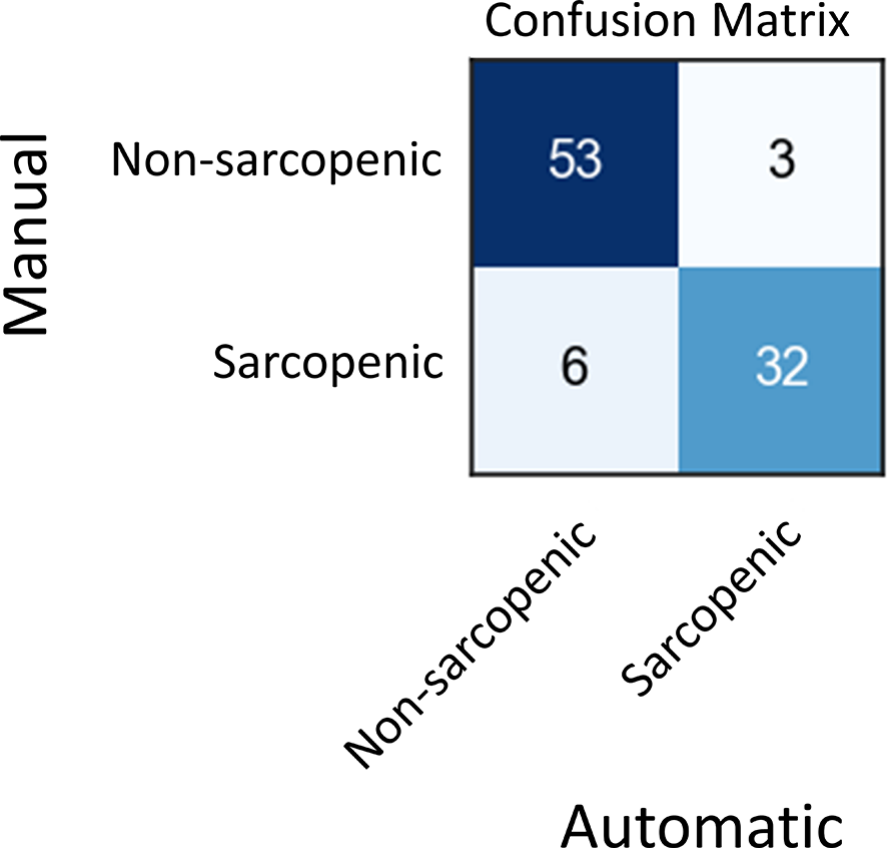
Sarcopenia classification based on manual and automatic SM area.

Skeletal muscle index (SMI) at L3, which is calculated by dividing skeletal muscle area by the height of patient squared, is a well-known surrogate for sarcopenia in cancer. In the test set, 35 patients out of 42 had recorded height and weight information, which we used to calculate SMI on each of their images. Then, we compared the SMI values based on manual and automated SM area for these patients (**Figure S6**, **Supplementary Table 3**). Finally, we use these SMI values to classify scans into sarcopenic and non-sarcopenic groups (**Figure 5**). Sarcopenic patients were classified based on reference values from Martin et al. (32). Sarcopenia was defined as SMI < 43 cm^2^/m^2^ in men with a body mass index (BMI) < 24.9 kg/m^2^ and < 53 cm^2^/m^2^ in men with a BMI > 25 kg/m^2^; and < 41cm^2^/m^2^ in women of any BMI. Out of 94 CT scans in the validation set for which we had the required clinical data, 85 were correctly classified as sarcopenic or not by automatic SM contours. The positive and negative predictive values shown in **Figure 5** resulted in a sensitivity of 84% and a specificity of 95%.

## Discussion

In this study, we present an ensemble model of 2.5D CNNs to automatically segment skeletal muscle on low quality CTs acquired in PET/CT studies, and investigate its qualitative and quantitative accuracy and precision to measure SM area and detect sarcopenia in NSCLC patients. It is widely recognized that robust measures of skeletal muscle mass are often challenging to use in clinical practice due to cost, time and the training required (33). This results in a tendency to use subjective assessments which have demonstrated inaccuracy in identifying sarcopenia (34).

The variation between the model and manual segmentation was similar to that measured between multiple observers; Perthen et al. (35) quantified inter-observer variation for L3 skeletal muscle area as delineated by three radiologists. The mean absolute difference between any two observers was up to 2.69 cm^2^. In our data set we achieved a mean absolute difference between two observers of 3.55 cm^2^ and a mean absolute difference between the manual and automated contours of 3.69 cm^2^. Further, the mean Dice score between our two observers was 0.96, compared with the mean dice score for our automated contours with manual contours of 0.92. We achieved a sensitivity of 84% and specificity of 95% when classifying sarcopenic patients using automatic contours and using Alberta protocol based manual diagnosis as ground truths. These results indicate our model has the potential to facilitate large scale robust assessment of skeletal muscle from low quality CT scans in the research setting, as well as clinical practice to support early identification and intervention.

Despite promising results, our study has several limitations. In some cases, with very low SM area, the model tends to misclassify other organs as belonging to skeletal muscle. These results suggest that the CNN has not been trained with images that fully represent the diversity and heterogeneity of SM area. To potentially overcome this problem, the proposed model can be retrained with new images as they are being acquired. We also observed limited benefit of data augmentation apart from flipping and addition of Gaussian noise, which may suggest limited variability in the validation set. Further improvement for use in external data sets may be achieved with increased variability in the training image acquisition and reconstruction parameters, and inclusion of images from a wider range of institutions. However, we observed that these limitations did not impact the ability to provide correct sarcopenia classification. Further, these can be improved by incorporating user interaction to correct mislabeled sections of CT. A further systematic difference between the manual and automated segmentation occurred when the patient was scanned with arms down; the model mis-classified portions of the arm at the L3 level, as there were no patients in the training set who were scanned with arms down.

Our approach is only trained and validated on attenuation correction quality CTs, specifically those that were obtained as part of PET/CT studies since these will typically contain L3 in the scan range. Our model shows improved results on our validation data set compared with a 2D AI model trained specifically on higher quality diagnostic CT scans. This suggests a domain specific training is likely required for widespread applicability of such models. Further, our model was a 2.5D model, which may provide further improvements over the 2D model, in particular with organ-muscle interfaces that may not be visible in the selected slice for analysis. Specific image normalization methods and model parameter tuning are needed to extend our method to other modalities, including diagnostic quality CTs and magnetic resonance imaging (MRI). Potential improvement may be achieved through use of higher quality diagnostic CT scans.

Deep learning-based methods are showing the potential to reliably automate a number of rudimentary pattern recognition tasks. If coupled with other methods to localize to the appropriate L3 slice (36), there is a pathway to fully automate these measures for any patient receiving CT imaging. It is foreseeable that being able to track trends in body composition would have implications in management in a number of chronic diseases, which are regularly monitored through volumetric CT or MR imaging.

## Conclusion

We present an automated method to delineate skeletal muscle area at L3 region of attenuation correction CT scans acquired as part of PET/CT studies for patients with NSCLC. The proposed method can be used to classify sarcopenia with minimal manual intervention, which may be an efficient method in large studies. Further, the model can be potentially used in clinical practice to identify early sarcopenia in patients with lung cancer.

## Data Availability Statement

The original contributions presented in the study are included in the article/**Supplementary Material**. Further inquiries can be directed to the corresponding author.

## Ethics Statement

The studies involving human participants were reviewed and approved by Peter MacCallum Cancer Centre. Written informed consent for participation was not required for this study in accordance with the national legislation and the institutional requirements.

## Author Contributions

All authors listed have made a substantial, direct and intellectual contribution to the work, and approved it for publication.

## Funding

This work was supported by the Peter MacCallum Cancer Centre Foundation.

## Conflict of Interest

The authors declare that the research was conducted in the absence of any commercial or financial relationships that could be construed as a potential conflict of interest.
