# Supplementary material for: A Deep Learning Model to Automate Skeletal Muscle Area Measurement on Computed Tomography Images
Source: Front Oncol. 2021 May 7;11:580806. doi: 10.3389/fonc.2021.580806 (PMC8138051; doi:10.3389/fonc.2021.580806)
Supplement: Supplementary file 1 [file DataSheet_1.pdf]

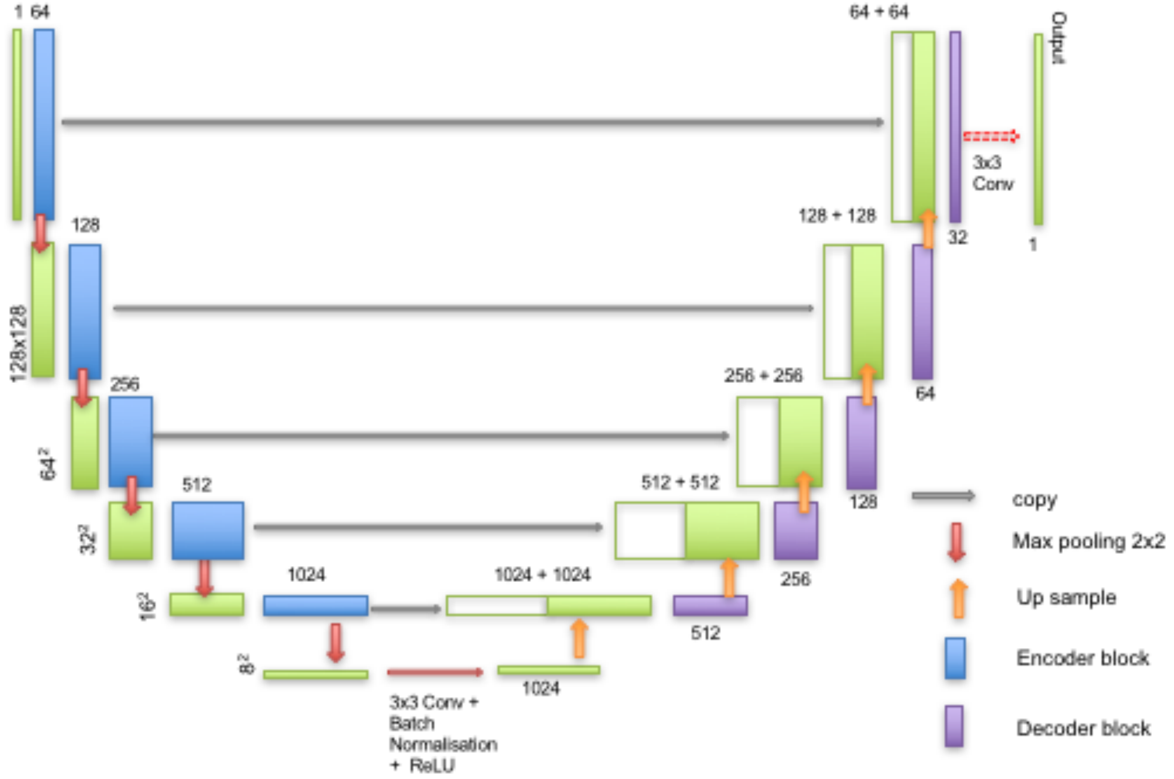

Figure S 1: 2D U-Net architecture

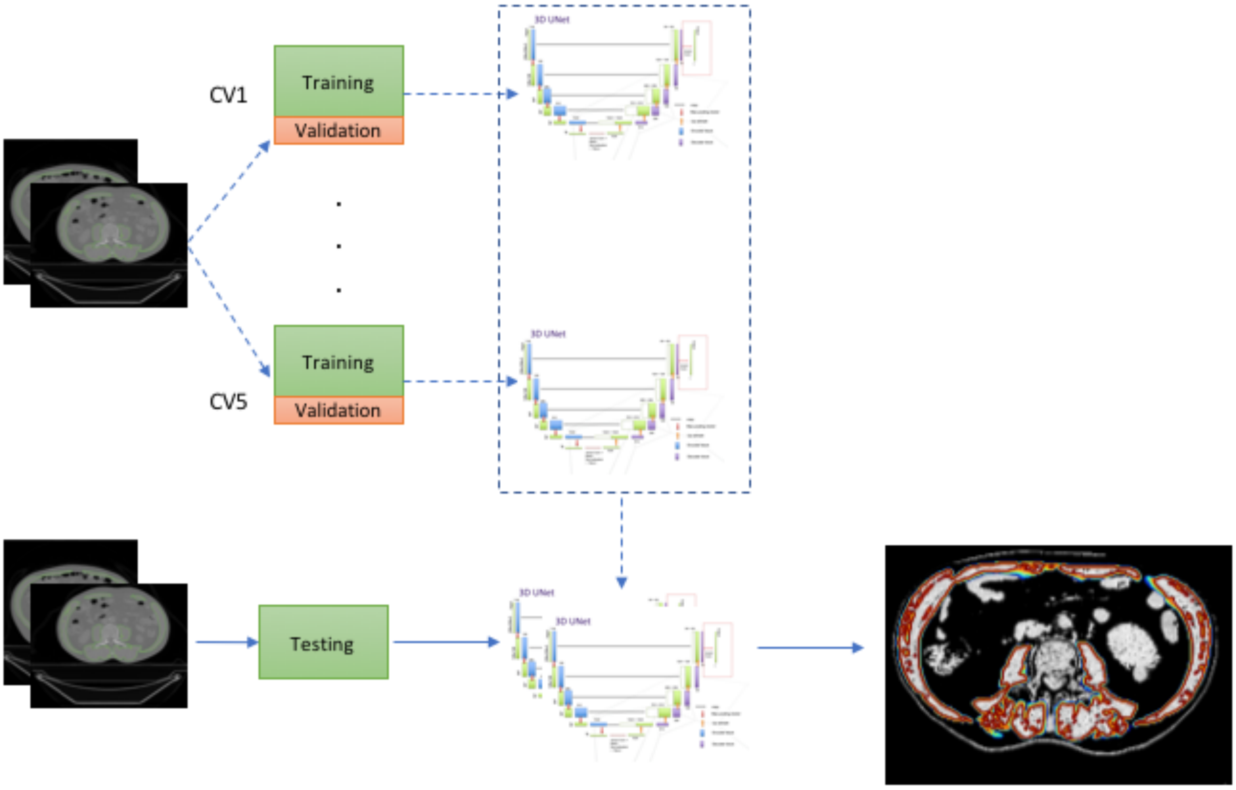

Figure S 2: 5-fold cross validation and ensemble learning model.

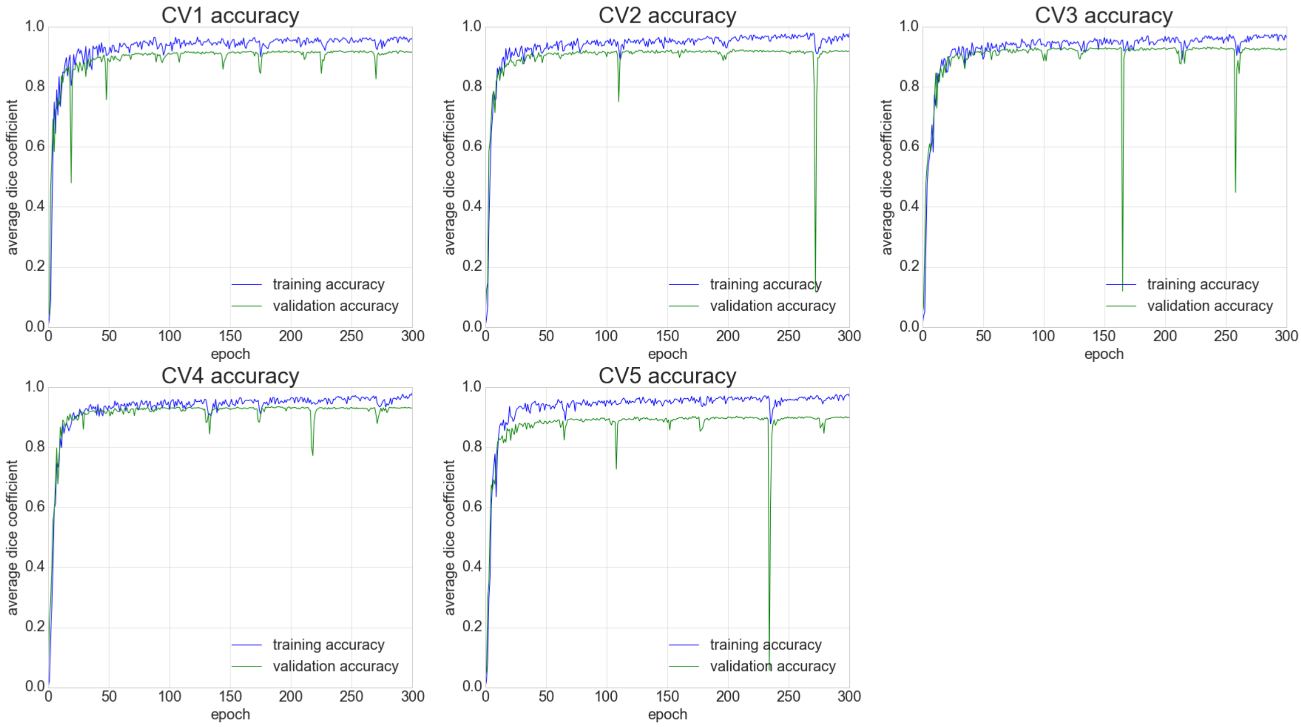

Figure S 3: Training and validation accuracies of the model for the 300 epochs.

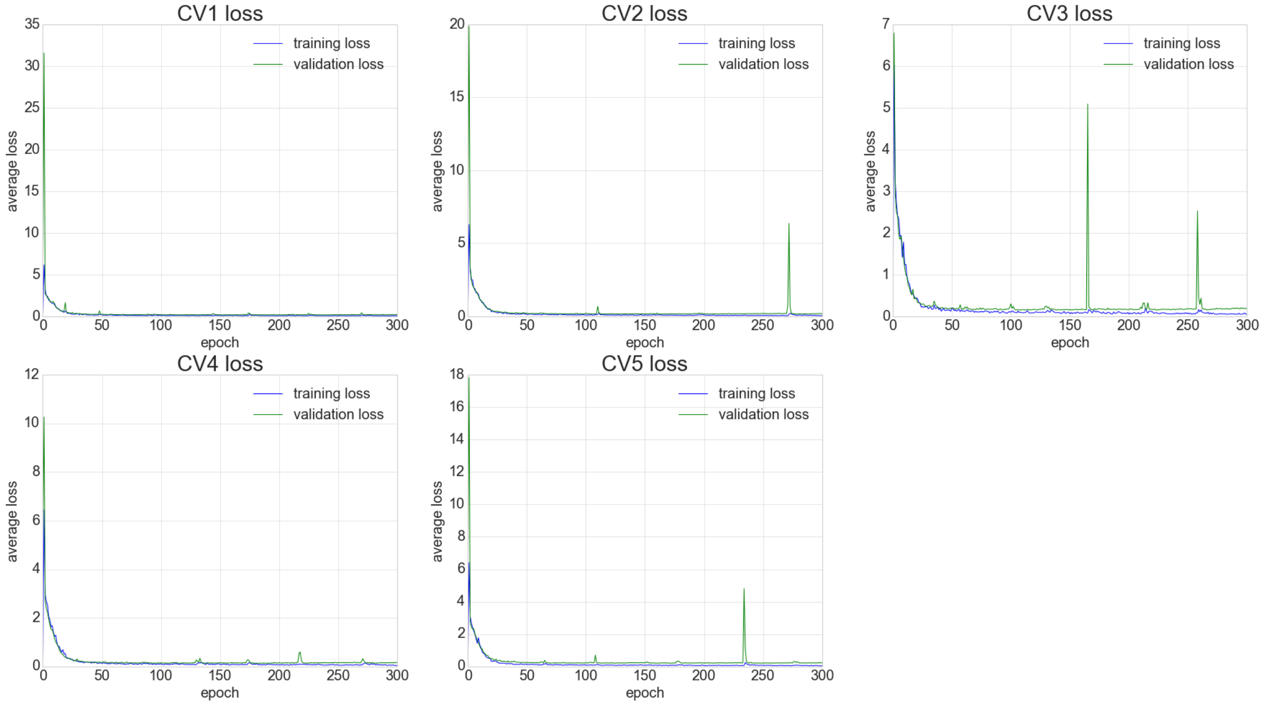

Figure S 4: Training and validation losses of the model for the 300 epochs.

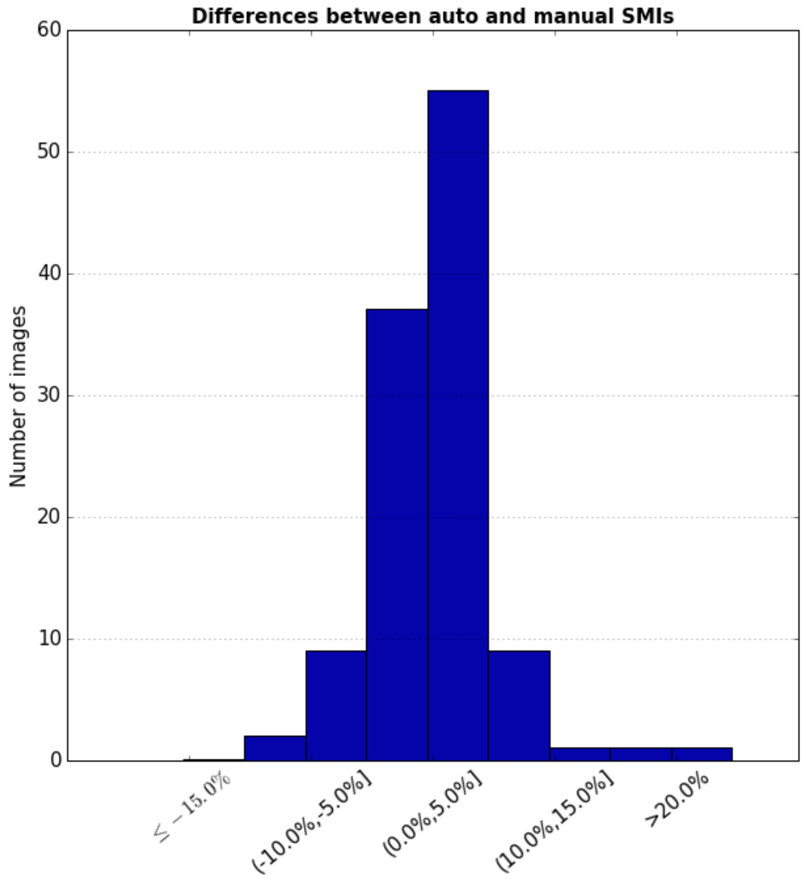

Figure S 5: Histogram of percentage error between manual and automated contours based on average probability maps.

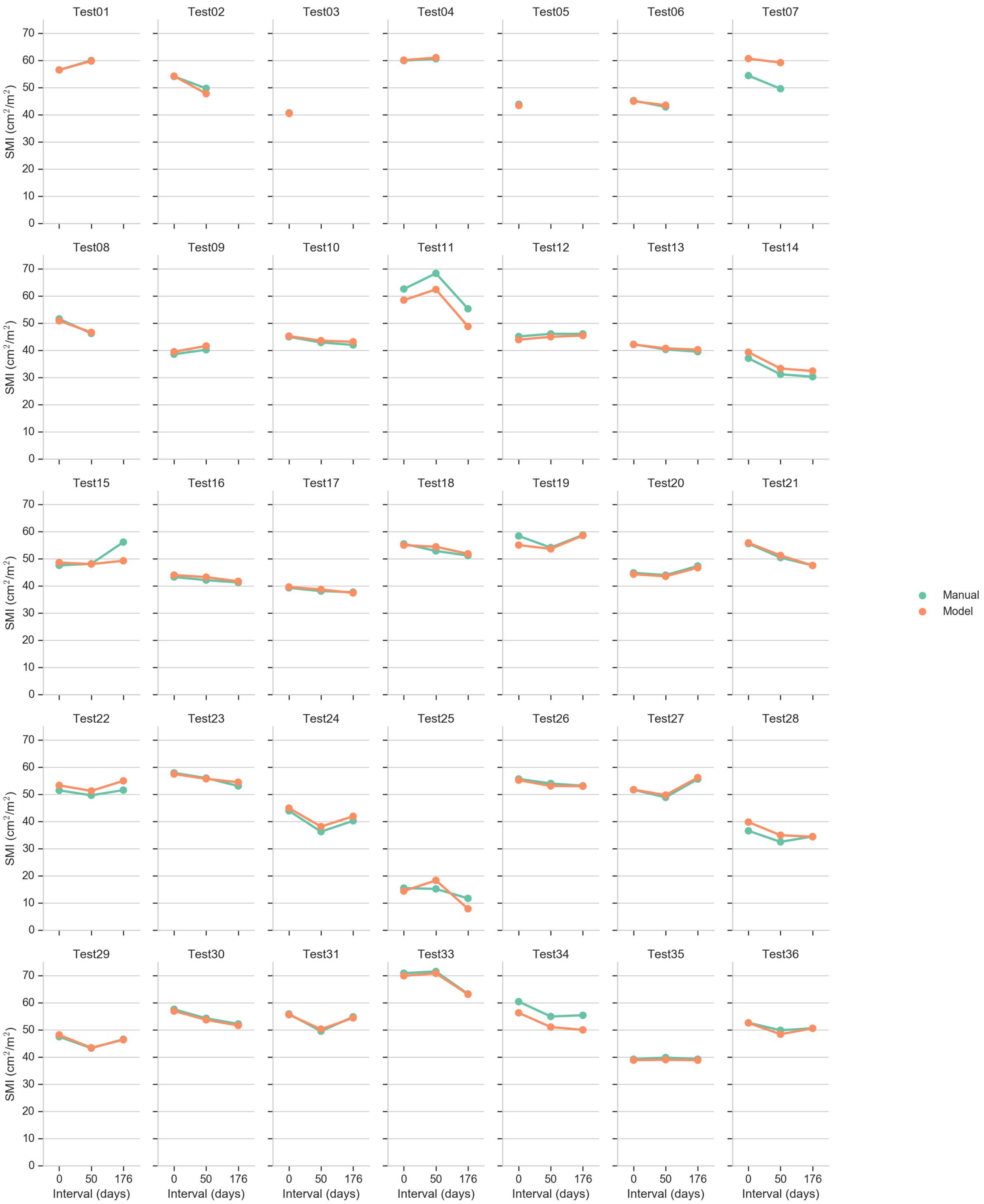

Figure S 6: Manual Vs automatic SMI
